# Evaluating citizen science data for forecasting species responses to national forest management

**DOI:** 10.1002/ece3.2601

**Published:** 2016-12-20

**Authors:** Louise Mair, Philip J. Harrison, Mari Jönsson, Swantje Löbel, Jenni Nordén, Juha Siitonen, Tomas Lämås, Anders Lundström, Tord Snäll

**Affiliations:** ^1^ Swedish Species Information CentreSwedish University of Agricultural Sciences (SLU)UppsalaSweden; ^2^ Department of Environmental System AnalysisInstitute of GeoecologyTechnical University BraunschweigBraunschweigGermany; ^3^ Department of Research and CollectionsNatural History MuseumUniversity of OsloOsloNorway; ^4^ Norwegian Institute for Nature ResearchOsloNorway; ^5^ Natural Resources Institute FinlandVantaaFinland; ^6^ Department of Forest Resource ManagementSwedish University of Agricultural Sciences (SLU)UmeåSweden

**Keywords:** deadwood‐dependent fungi, forestry, global biodiversity information facility, habitat change, land use change, opportunistic data, volunteer recording

## Abstract

The extensive spatial and temporal coverage of many citizen science datasets (CSD) makes them appealing for use in species distribution modeling and forecasting. However, a frequent limitation is the inability to validate results. Here, we aim to assess the reliability of CSD for forecasting species occurrence in response to national forest management projections (representing 160,366 km^2^) by comparison against forecasts from a model based on systematically collected colonization–extinction data. We fitted species distribution models using citizen science observations of an old‐forest indicator fungus *Phellinus ferrugineofuscus*. We applied five modeling approaches (generalized linear model, Poisson process model, Bayesian occupancy model, and two MaxEnt models). Models were used to forecast changes in occurrence in response to national forest management for 2020‐2110. Forecasts of species occurrence from models based on CSD were congruent with forecasts made using the colonization–extinction model based on systematically collected data, although different modeling methods indicated different levels of change. All models projected increased occurrence in set‐aside forest from 2020 to 2110: the projected increase varied between 125% and 195% among models based on CSD, in comparison with an increase of 129% according to the colonization–extinction model. All but one model based on CSD projected a decline in production forest, which varied between 11% and 49%, compared to a decline of 41% using the colonization–extinction model. All models thus highlighted the importance of protected old forest for *P. ferrugineofuscus* persistence. We conclude that models based on CSD can reproduce forecasts from models based on systematically collected colonization–extinction data and so lead to the same forest management conclusions. Our results show that the use of a suite of models allows CSD to be reliably applied to land management and conservation decision making, demonstrating that widely available CSD can be a valuable forecasting resource.

## 1 Introduction

Species distribution models (SDMs) have been extensively applied in forecasting species responses to future habitat and climate change (Elith & Leathwick, 2009). The temporal and spatial extent of such studies can be expanded through the increasingly popular use of citizen science data (CSD) (Devictor, Whittaker, & Beltrame, 2010). CSD provide an inexpensive source of species observation data, particularly as the online collation of data is becoming common practice for many regions of the world (Silvertown, 2009). This greatly expands the potential scope of SDM forecasting studies. Forecasts can provide valuable insights into possible future conditions, allowing land use managers and conservationists to make informed decisions (Mouquet et al., 2015).

A drawback of CSD is that they are frequently presence‐only observations, which cannot be modeled using established presence–absence frameworks such as generalized linear models (GLMs). New methods have therefore been developed specifically to model presence‐only data; foremost of these is MaxEnt (Phillips, Anderson, & Schapire, 2006). MaxEnt has been shown to outperform other methods when predicting species’ distributions and has been extensively tested against presence–absence methods such as GLMs (e.g., Elith et al., 2006). MaxEnt has been widely applied to CSD and used to address a diverse range of topics, including conservation applications (Elith et al., 2011). Yet, MaxEnt has often been misunderstood or misused (Yackulic et al., 2013). Therefore, any inferences made from model projections must be carefully assessed, particularly in a management context.

A second drawback is that CSD often suffer from spatial recording biases (Dickinson, Zuckerberg, & Bonter, 2010). Volunteer recorders may disproportionately visit sites close to home or roads, or may favor species‐rich habitats (Dennis & Thomas, 2000). If observation data are presence‐only, then separating out species–habitat associations from volunteer‐habitat preferences can be difficult (Barbosa, Pautasso, & Figueiredo, 2013). Spatial or environmental filtering of records can reduce bias and improve model performance (Boria, Olson, Goodman, & Anderson, 2014); however, such methods involve throwing away data. Alternatively, spatial recording bias can be explicitly modeled using a small amount of presence–absence data (Fithian, Elith, Hastie, & Keith, 2015). This reduces the investment required in obtaining presence–absence data while making use of extensive presence‐only datasets. This approach performed well on one species group (Fithian et al., 2015), but has yet to be widely tested.

Thirdly, the imperfect detection of species in the field is a general feature of observation data, yet is rarely accounted for in SDMs (Lahoz‐Monfort, Guillera‐Arroita, & Wintle, 2014). The detectability of a species (the probability that an individual is observed where present) may vary among sites and/or over time (van Strien, van Swaay, & Kery, 2011). In the context of citizen science, detection may also vary among recorders due to differing identification skills or search effort. We henceforth use the term “occupancy model” for joint modeling of occurrence and detectability (MacKenzie et al., 2002). Occupancy models were initially developed to account for imperfect detection using repeat‐survey data, but have recently been applied to ad hoc CSD, successfully recovering expected trends in species’ distributions (van Strien, van Swaay, & Termaat, 2013). Moreover, occupancy models identified biologically reasonable species–habitat associations when applied to spatially biased data, in contrast to conventional regression models (Higa et al., 2015). The application of occupancy models to spatially biased and/or ad hoc data is as yet very limited, however, and further testing is required to determine whether inferences from a diversity of datasets are reliable.

There are thus a broad variety of modeling approaches available and previous work has concluded that no single method consistently produced the most accurate results (Qiao, Soberón, & Peterson, 2015). Moreover, different approaches to deal with recording biases can produce different conclusions (Isaac, van Strien, August, de Zeeuw, & Roy, 2014). A further source of variation stems from the increasingly popular technique of combining correlative and mechanistic components in species distribution modeling. The combination of correlative and mechanistic components, such as physiological constraints or population dynamics, has been advocated to improve the biological realism of models (Kearney & Porter, 2009). However, the inclusion of mechanisms can quantitatively change projected trends (Swab, Regan, Matthies, Becker, & Bruun, 2015), implying yet another source of variation among methods. Therefore, it may in fact be preferable to apply multiple methods in order to address sources of uncertainty (Qiao et al., 2015).

A limitation of many modeling studies that apply CSD is the lack of validation against independent models based on systematically collected data. If CSD are to be widely applied in areas such as land management and conservation decision making, then the ability of models based on CSD to produce forecasts that are congruent with forecasts from models based on systematically collected data should be demonstrated. Congruence would provide confidence in applying cheap, widely available CSD to a range of forecasting questions, which would increase the scope of forecasting studies and avoid the need for costly, time‐consuming data collection by experts.

In this study, we aimed to assess the reliability of species occurrence forecasts from models based on CSD. We tested whether five different occurrence models based on open access CSD produced forecasts that were congruent with forecasts from a dynamic model based on colonization–extinction data that were systematically collected by experts. We thus compared forecasts from models based on differing quality of data (in terms of citizen scientist versus expert collection) and differing biological information content (occurrence CSD versus dynamic colonization–extinction data). We projected changes in the occurrence of *Phellinus ferrugineofuscus*, an old‐forest indicator fungus, in response to national forecasts of forest management in Sweden. All five species distribution models based on CSD utilized presence‐only and/or presence–absence data collected by volunteer recorders and were selected to encompass a diverse range of data requirements and assumptions about recording biases.

## 2 Methods

### 2.1 Study species

*Phellinus ferrugineofuscus* is a polyporus species associated with Norway spruce, *Picea abies*. Polyporus fungi are important deadwood decomposers and many species are negatively affected by forest management (Nordén, Penttilä, Siitonen, Tomppo, & Ovaskainen, 2013). The occurrence of *P. ferrugineofuscus* is determined by deadwood availability and connectivity old spruce‐dominated forest (Jönsson, Edman, & Jonsson, 2008). *Phellinus ferrugineofuscus* is classified as near threatened (NT) in Sweden due to forestry (Artdatabanken, 2015). It has been widely used as an old‐forest indicator species in nature conservation inventories in the Nordic countries (Niemelä, 2005). *Phellinus ferrugineofuscus* is easy to find and identify in the field.

### 2.2 Citizen science species observation data

**Figure 1:**
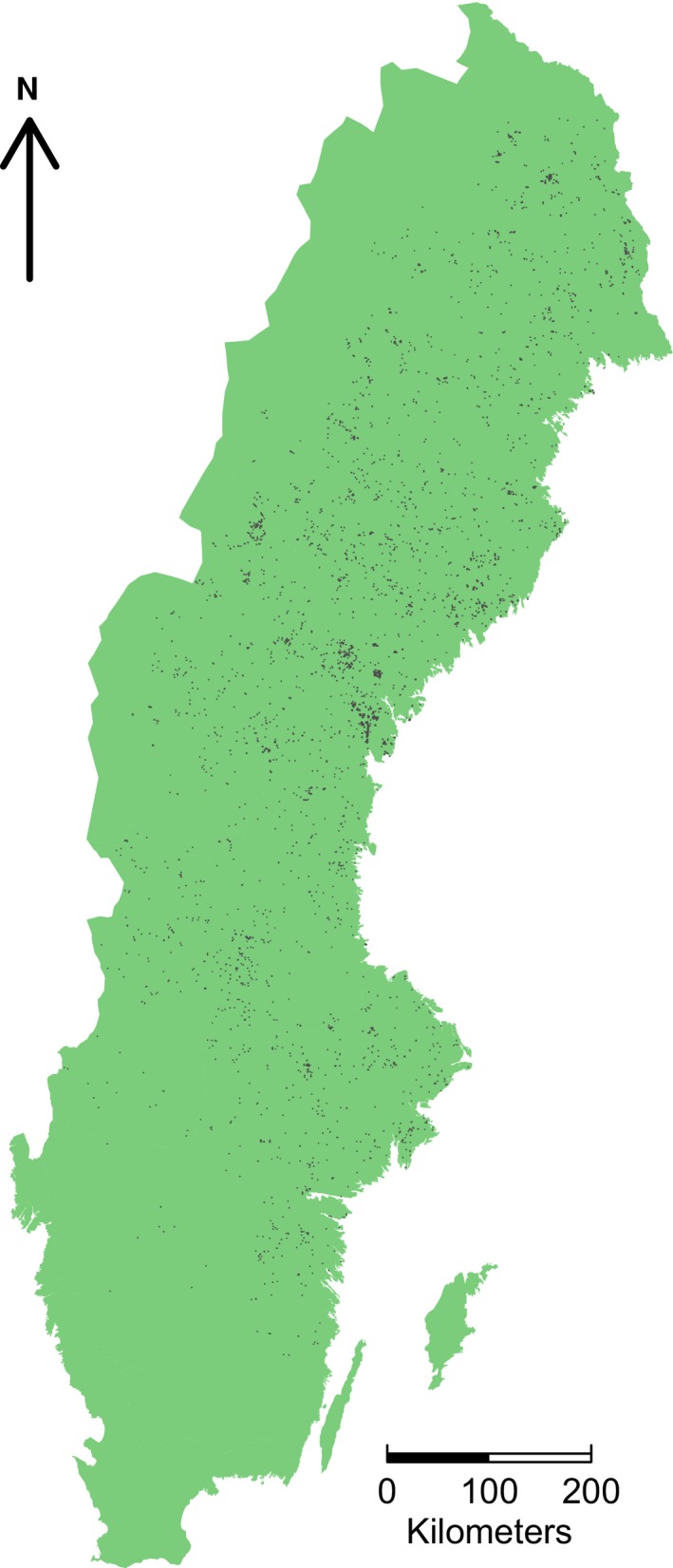
Observed 100 m grid cell resolution occurrences of *Phellinus ferrugineofuscus* 2000–2013 (*N* = 5,317) obtained from Swedish Lifewatch (analysisportal.se)

Citizen science data for *P. ferrugineofuscus* were downloaded from the Swedish open access Lifewatch website (www.analysisportal.se) for the period 2000–2013 at the 100 m grid cell resolution. Observations were presence‐only, and the species was recorded in 5,317 cells (Figure 1). The Lifewatch website is a portal that compiles observation data from multiple sources. The primary source for fungal observations is the Swedish Species Observation System (www.artportalen.se). Data uploaded to the Species Observation System come from many different recorders ranging from amateur enthusiasts to trained field workers carrying out inventories for forestry companies. Data may be complete species checklists or single species observations; however, as recorders are not required to register species absences, this information is unknown.

To obtain a presence–absence dataset for *P. ferrugineofuscus*, we interviewed recorders of wood‐dependent fungi. Each recorder was asked the same questions about their field methods. If field searches were thorough and consistent (see Appendix S1 in Supporting Information), then observation records from that recorder were compiled to create a presence–absence dataset. Among these, the presence of species other than the target species was taken to indicate the absence of the target species. Data from eight recorders were used covering 15,508 grid cells (Appendix S1).

### 2.3 Environmental data

We hypothesized that *P. ferrugineofuscus* occurrence probability increased with living spruce volume and forest stand age. Forest data were based on estimates which combine satellite images and ground‐truthing; “kNN‐Sweden” (http://skogskarta.slu.se; Reese et al., 2003; for details, see Appendix S2). During model development, it became clear that recording effort was biased toward older forest. Therefore, forest age was excluded in order to avoid modeling recording bias rather than species occurrence.

The kNN data were also used to test the hypothesis that species occurrence increased with connectivity to old forest, which reflects the potential dispersal sources for the species in the surrounding landscape. We used a connectivity calculation adapted from Nordén et al. (2013) (detailed in Appendix S2). We tested three values for the dispersal parameter representing a mean dispersal distance of 1, 5, and 10 km.

We hypothesized that *P. ferrugineofuscus* occurrence was negatively related to temperature and precipitation, given the northern boreal distribution of the species. We also hypothesized that there was an interactive effect as the effect of high water availability on fungal activity is lower at colder temperatures due to reduced metabolic rates (Boddy et al., 2014). Gridded meteorological data were obtained from the EURO4M Mesan dataset (Landelius, Dahlgren, Gollvik, Jansson, & Olsson, 2016). We used mean annual temperature and seasonally accumulated precipitation from May to November, both averaged over the period 1989–2010 (see Appendix S2 for details). This time frame includes the 10 years prior to the species observation data as fruiting bodies observed from 2000 onwards may reflect colonization several years earlier.

We calculated a wetness index and a variable which reflected the steepness and orientation of a grid cell using a digital elevation map (Swedish land survey service; www.lantmateriet.se; calculations in Appendix S2). The hypothesis was peak occurrence at intermediate wetness, which represents the optimum conditions for the species’ primary habitat. For the variable reflecting steepness and orientation, we hypothesized a linear relationship reflecting increased occurrence on steeper, north‐facing slopes due to lower sun exposure.

One of the modeling approaches we applied accounted for spatial biases in the collection of presence‐only data (Fithian et al., 2015). We used the variables population density (number of people per km^2^ in 2010; Statistics Sweden, www.scb.se), log population density, distance to small roads, distance to main roads, distance to the five largest cities, distance to all cities, and distance to towns (road and urban area data from the Swedish land survey service). All variables were transformed from polygon data to 100 m grid cells. We tested for both linear and quadratic effects of each bias variable.

### 2.4 Occurrence models based on citizen science data

The complexity of models was constrained to improve comparative ability among models, to allow evaluation of the biological plausibility of the species’ response curves, and to avoid overfitting (Merow et al., 2014). To facilitate assessment of the relative importance of covariates, all variables were standardized (division with the standard deviation) prior to modeling. All modeling based on CSD was carried out at the 100 m grid cell resolution and the occurrence data were utilized as a single snapshot.

#### 2.4.1 GLM

A generalized linear model with a binomial distribution and logit link was fitted to the presence–absence data. We first fitted a model using living spruce volume as the explanatory variable. Model complexity was then assessed using AIC (Burnham & Anderson, 2002) to ensure that model fit was improved with the inclusion of further covariate or interaction terms, see *Environmental data* above. Models were fitted using R version 3.1.0 (R Core Team, 2014).

#### 2.4.2 MaxEnt

MaxEnt is a maximum entropy model which makes use of species presence‐only observations and a background sample (Elith et al., 2011; Phillips et al., 2006). The background sample may also be referred to as “pseudo‐absence” data. We used two approaches to obtain the background sample. Firstly, we sampled 40,000 grid cells randomly from the study area, excluding cells with presence‐only records of the focal species. Secondly, in order to account for recording biases, we applied the target‐group background (TGB) method (Phillips & Dudik, 2008), where background cells were selected based on the presence of species with similar recording biases (but not the focal species). We selected wood‐dependent fungal species (*N* = 202; Stokland & Meyke, 2008) as the target group. This gave 34,430 background cells (downloaded from Swedish Lifewatch for 2000–2013 at 100 m resolution).

In order to prevent the inclusion of spurious interactions or quadratic terms with no biological justification, we created all interactions and quadratic terms and entered them into MaxEnt as so‐called linear features. All other MaxEnt features were switched off (Phillips & Dudik, 2008). Variable selection was carried out by maintaining only the covariates which had an importance or contribution greater than zero. AUC was calculated on the presence–absence data to ensure that no loss in predictive ability occurred when variables were removed. Models were fitted using MaxEnt version 3.3.3 run from R using the *dismo* package version 1.5 (Hijmans, Phillips, Leathwick, & Elith, 2014).

#### 2.4.3 PA/PO model

We also applied an inhomogeneous Poisson point‐process model which combines presence‐only and presence–absence species’ observation data (termed here “PA/PO model”; Fithian et al., 2015). The approach models species occurrence against environmental variables while explicitly modeling spatial bias in recording effort, by combining a species occurrence component and a recording bias component. The model requires presence‐only data for multiple species, a small sample of presence–absence data, and a background sample.

We used presence‐only and presence–absence data for our study species and six other spruce‐associated deadwood‐dependent fungi (*Amylocystis lapponica*,* Fomitopsis rosea*,* Leptoporus mollis*,* Phellinus chrysoloma*,* Phellinus nigrolimitatus*, and *Phlebia centrifuga*). For the background sample, we randomly sampled 40,000 cells across the study area. We tested the environmental and bias variables described in *Environmental data* above. Variable selection was based on AIC for *P. ferrugineofuscus*. Models were fitted in R using the package *multispeciesPP* version 1.0.

#### 2.4.4 Occupancy model

Estimating species detectability using occupancy modeling relies on data from repeat visits to sites within a closed period. We established a detection/nondetection dataset for *P. ferrugineofuscus* using the presence‐only citizen science data. We first identified other old‐forest indicator species of deadwood‐dependent fungi which, based on our knowledge, citizen scientists interested in *P. ferrugineofuscus* were highly likely to also search for and record when found (*N* = 35; see Appendix S3). We used detections of indicator species other than our focal species to indicate the nondetection of the focal species. A small proportion of grid cells had two or more species observation records occurring on different days within the same calendar year, and we utilized these observations as repeat‐visit data. We used a calendar year as the definition of a closed period as the species’ fruiting body life span is 1–2 years. The data consisted of 29,615 grid cells, of which 807 grid cells received two or more visits (of these, maximum number of visits = 7, median = 2).

We formulated the occupancy model in a Bayesian framework. The probability of occurrence and the probability of detection were modeled as a logistic function, essentially as in Kéry, Gardner, and Monnerat (2010). Observed data are a result of the interaction between the true occurrence and the detectability of the species. True occurrence was modeled as a function of the environmental variables. Detectability was assumed to vary among recorders (and therefore to vary among sites and visits depending on the recorder present) and was modeled against the total number of days each individual recorder had submitted records of wood‐living indicator species during the study period. For a discussion of the detectability variables considered, see Appendix S4.

Variable selection for species occurrence was based on the posterior distributions of the parameters (the use of DIC is not appropriate for mixture/hierarchical models; Hooten & Hobbs, 2014). If the 95% credible interval of the parameter estimate did not include zero, then the variable was considered to be significant. We started with a model which included living spruce volume as the explanatory variable for occurrence and an intercept‐only detection model. Complexity was increased by adding one variable at a time and assessing significance. Once the species occurrence model was established, the detectability model was fitted. The models were fitted using OpenBUGS (Lunn, Spiegelhalter, Thomas, & Best, 2009) through R using the packages *R2OpenBUGS* and *BRugs*. We ran two chains with 80,000 iterations thinned by two, after a burn‐in of 20,000 iterations. The BUGS code for the final model is given in Appendix S5.

### 2.5 Colonization–extinction model based on systematically collected field data

Occurrence models based on CSD were compared against a dynamic model fitted to systematically collected data on colonization–extinction events (Harrison, P.J, Mair, L, Nordén, J, Siitonen, J, Lundström, A, Kindvall, O, Snäll, T, in preparation). To obtain colonization–extinction data, we conducted resurveys in 2014 of 174 forest stands in Finland that were initially surveyed in 2003–2005 (Nordén et al., 2013). In both time periods, we inventoried all deadwood objects with a diameter at breast height (DBH) ≥5 cm and length ≥1.3 m within a fixed survey plot (usually 20 m × 100 m) inside each stand. Deadwood characteristics (used as explanatory variables in addition to those described in *Environmental data* above) and polypore presences were recorded.

We modeled the cut and noncut stands separately. We used forward stepwise model selection and variables were retained based on the posterior distributions of the parameters. We first define *Z*_*j*,*t*_ as the true occupancy state of plot *j* during survey period *t*. We assume that *Z*_*j*,*t*_ *~* Bernoulli(ψ_*j*,*t*_). For the second survey period:

$$\psi_{j,t}=\left(1-Z_{j,t-1}\right)c_{j,t}^{*}+Z_{j,t-1}\left(1-e_{j,t}^{*}\right)$$

where $c_{j,t}^{*}$ and $e_{j,t}^{*}$ give, respectively, the colonization and extinction probabilities, which have been offset to correct for the different numbers of years between the surveys and the different plot areas, such that:

$$c_{j,t}^{*}=1-{\left(1-c_{j,t}\right)}^{n_{j,t}a_{j,t}}$$

$$e_{j,t}^{*}=1-{\left(1-e_{j,t}\right)}^{\frac{n_{j,t}}{a_{j,t}}}$$

where *n*_*j*,*t*_ gives the number of years between the surveys divided by 10 (i.e., scaled by the typical number of years), and *a*_*j*,*t*_ gives the plot area divided by 0.2 (i.e., scaled by the typical plot size in hectares). If the forest in the plot had been clear‐cut (either before the first survey or between the two survey events), then cloglog(*c*_*j*,*t*_) = δ_1_ and cloglog(*e*_*j*,*t*_) = $\varepsilon_{1}$. We chose to use the complementary log–log link function, cloglog, as due to its asymmetrical nature it is better suited than the more conventional logistic link function to cases where the probabilities are very large or very small. If the forest in the plot had not been clear‐cut, we assumed that:

$$\text{cloglog}\left(c_{j,t}\right)=\delta_{2}+\sum_{l}\beta_{l}X_{l,j,t}$$

and that cloglog(*e*_*i*,*j*,*t*_) = $\varepsilon_{2}$. Due to data limitations, we could not include covariates in the models for the extinction probabilities or the colonization probability on clear‐cut cells (intercept‐only models were used in these cases). The *l* covariates used in the model for the colonization probability are given by *X*_*l*,*j*,*t*_ with corresponding parameter values β_*l*_. Finally, we define *Y*_*j*,*t*_ as the observed occupancy state of plot *j* during survey period *t*. For the observation model, we assume that *Y*_*i*,*j*,*t*_ ~ Bernoulli(*Z*_*j*,*t*_ *p*) where *p* gives the detection probability. This detection probability was estimated as 0.9 based on an intensive control study. No colonization events occurred on cut sites and so their colonization probability was set to zero. In order to initialize the models used to simulate the future dynamics of the polypore species, we used a model fitted to the occurrence data from 2014.

### 2.6 Temporal forecasts of species occurrence in response to forest management

In order to test whether the occurrence models based on CSD produced forecasts that were congruent with forecasts from the colonization–extinction model based on systematically collected dynamics data, we used the models to project species occurrence in response to a forest management scenario. Forest projection data were available from the Swedish nationwide Forest Scenario Analyses 2015 (FSA 15; Claesson, Duvemo, Lundström, & Wikberg, 2015; Eriksson, Snäll, & Harrison, 2015). Using the Heureka system (Wikström et al., 2011), projections were made for the National Forestry Inventory (NFI) plots (Fridman et al., 2014) for every fifth year from 2020 to 2110. We used data for a total of 17,383 NFI plots from the whole boreal region of Sweden (160,366 km^2^ of productive forest). Data on projected changes in living and deadwood spruce volume and forest age were available (for data details see Appendix S6 and for calculation of connectivity see Appendix S7). We used a scenario which assumes that 84% of the land is used for wood production and 16% is set‐aside from forestry. The aim of set‐aside forest is to improve biodiversity conservation within the forested landscape.

Projections of species response to forest management were based on a space–time substitution, such that we projected the occurrence of the species across the NFI plots at each time step, and so obtained the change in species occurrence over time. The procedure was as follows. Separately for each of the models, we predicted the probability of species’ occurrence at each NFI plot for each time step. Mechanistic assumptions were then incorporated into the projections. The species could not occur where no deadwood was present (it is a deadwood‐dependent species), or where forest age was 25–64 years (due to deadwood turnover on cut sites; see Appendix S8 for details). The values predicted at each plot were then scaled to reflect the proportion of the total country that each plot represents (density of plots varies across the country and thus the area that each plot represents varies). Scaled probabilities were summarized across the whole region and separated into production and set‐aside forest. Temporal projections using the models based on CSD were compared against projections using the colonization–extinction model. We also calculated the relative change in species occurrence over time. Finally, we averaged projections of relative change across all five models based on CSD in order to test an ensemble modeling approach.

We investigated the sensitivity of the results to the mechanistic assumptions outlined above. We compared projections from the models based on CSD including (i) no mechanistic assumptions; (ii) the forest age threshold assumption alone; (iii) the deadwood presence assumption alone; and (iv) both mechanistic assumptions together.

### 2.7 Spatial prediction of current occurrence

To assess the spatial accuracy of predictions of current species’ occurrence from the models based on CSD, we used block cross‐validation and calculated the area under the receiver operating curve (AUC; see Appendix S9 for details). We also used the models to predict the current distribution of *P. ferrugineofuscus* in Sweden at the 10 km grid cell resolution. Species probabilities of occurrence were predicted across the 100 m resolution sample of random background points and aggregated to 10 km resolution using the mean. We applied the mechanistic assumption relating to forest age, but could not apply the deadwood assumption as no national GIS layer on deadwood occurrence exists. Maps were compared visually.

## 3 Results

### 3.1 Temporal projections: forest management scenario

**Figure 2:**
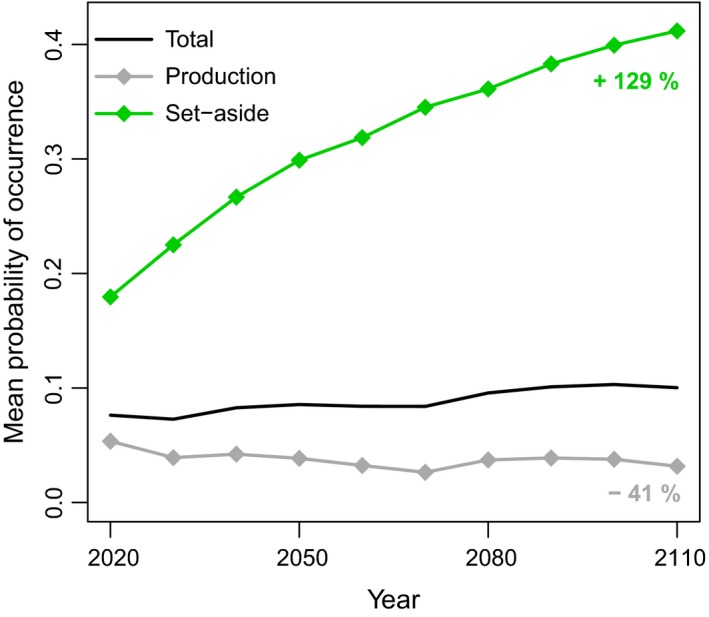
Forecasts of mean probability of *Phellinus ferrugineofuscus* occurrence in response to projected forest management over the coming century from the colonization–extinction model based on systematically collected data. Mean probability of occurrence is presented for all forest and for production and set‐aside forest separately. The relative changes in probability of occurrence (%) from 2020 to 2110 are given for set‐aside and production forest

**Figure 3:**
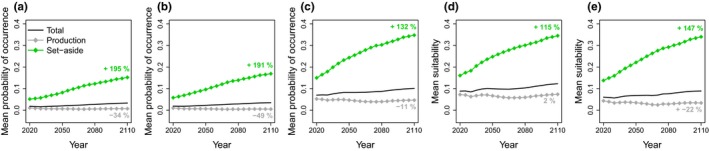
Forecasts of mean probability of *Phellinus ferrugineofuscus* occurrence (or suitability) in response to projected forest management over the coming century using models based on citizen science data. Models used were (a) GLM; (b) PA/PO model; (c) occupancy model; (d) MaxEnt random background; and (e) MaxEnt TGB. Mean probability of occurrence is presented for all forest and for production and set‐aside forest separately. The relative changes in probability of occurrence (%) from 2020 to 2110 for each model type are given for set‐aside and production forest

Forecasts from the occurrence models based on CSD were generally congruent with forecasts from the colonization–extinction model based on systematically collected data (Figures 2 and 3). All models projected probability of occurrence of *P. ferrugineofuscus* (or suitability in the case of MaxEnt) to be lower in production forest than in set‐aside forest set‐aside (Figure 3). Probability of occurrence was projected to increase over time in set‐asides, but to decline in production forest according to all but one of the models based on CSD (MaxEnt TGB projected a slight increase).

Although all models projected comparable trends, different models projected different amounts of change over time. The increase from 2020 to 2110 in probability of occurrence in set‐asides varied between 115% and 195% among models based on CSDs, compared to an increase of 129% projected by the colonization–extinction model. In production forest, only the MaxEnt TGB model projected a slight increase in probability of occurrence of 2%, while the remaining models based on CSD projected declines of 11% to 49%. The colonization–extinction model projected a decline of 41%.

**Figure 4:**
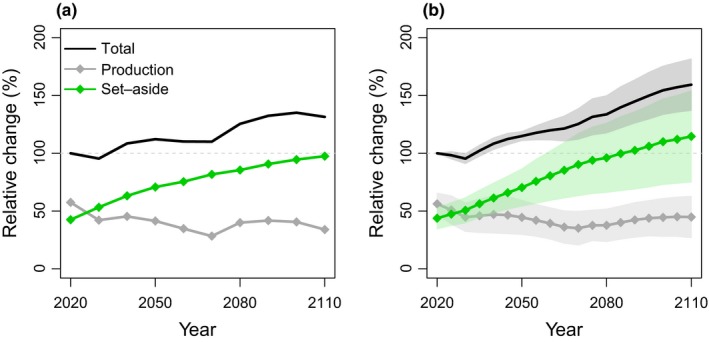
Forecasts of relative change in *Phellinus ferrugineofuscus* occurrence in response to projected forest management over the coming century from (a) the colonization–extinction model based on systematically collected data and (b) averaged projections from the models based on citizen science data (mean ± *SD*). Relative change is presented for all forest (“total”) and for production and set‐aside forest separately

Projected trends in relative change over time were very similar between the colonization–extinction model and the averaged models based on CSD, although the latter projected larger increases in set‐aside forest (Figure 4). Averaging across models based on CSD gave an increase of 162% in set‐asides and decline of 20% in production forest.

### 3.2 Spatial predictions: species distributions maps

Similar AUC scores on both training and withheld testing data were obtained for all models based on CSD (Appendix S9), suggesting that the different approaches all achieved good fits. The mean training AUC was 0.83–0.84 and mean testing AUC was 0.78–0.79.

**Figure 5:**
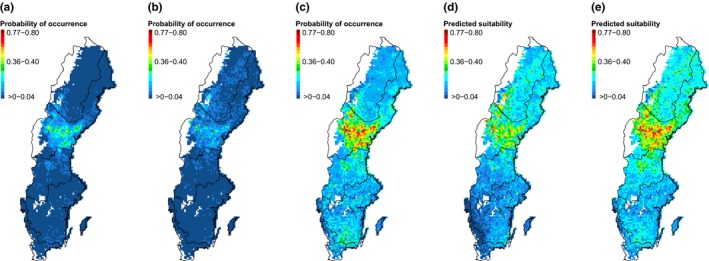
Maps of the predicted probability of *Phellinus ferrugineofuscus* current occurrence (or predicted suitability in the case of MaxEnt models) at 10 km grid cell resolution for (a) GLM, (b) PA/PO model, (c) occupancy model, (d) MaxEnt random background, and (e) MaxEnt TGB

All five approaches highlighted central Sweden as having the highest probability of *P. ferrugineofuscus* occurrence (Figure 5). The GLM, PA/PO model, and occupancy model differed in absolute probabilities, with the occupancy model predicting generally higher values. The MaxEnt model predictions of relative suitability were typically also higher values.

### 3.3 Key environmental variables in models based on citizen science data

Final models had varying structures but notable similarities (Appendix S10). All models identified living spruce volume as the variable with the strongest positive relationship with *P. ferrugineofuscus* occurrence. The variable with the second strongest and positive effect was connectivity. Fitted lines illustrating the effects of the four most important variables (spruce volume, connectivity, temperature, and precipitation) indicated that the MaxEnt TGB model identified a weaker effect of spruce volume relative to the other modeling approaches (Appendix S10).

The variables explaining spatial recording biases identified by the PA/PO model were population density and distance to small roads (Appendix S10). The recording bias was highest at intermediate densities (around 2220 people per km^2^), falling to very low recording probabilities at the extremes of population density. Recording bias was highest at short distances from small roads.

The sensitivity analysis showed that the overall probability of occurrence (or suitability) was reduced with the inclusion of mechanistic assumptions (Appendix S10). The inclusion of the deadwood presence assumption resulted in a greater reduction in probability of occurrence than inclusion of the forest age assumption. The inclusion of mechanistic assumptions resulted in both greater increases over time in set‐aside forest and more negative trends in production forest relative to projections that did not incorporate mechanistic assumptions.

### 3.4 Colonization–extinction model

From the Finnish plot‐level data, we observed nine extinction events (four on noncut sites and five on cut sites) and twelve colonization events (all of which occurred on the noncut sites). Only stand age was selected as the variable explaining the colonization probability of noncut sites (Harrison et al. in prep).

## 4 Discussion

Species distribution models built using citizen science data forecast changes in *P. ferrugineofuscus* occurrence in response to forest management that were qualitatively congruent with forecasts from a colonization–extinction model built using systematically collected data (Harrison et al. in prep). The five modeling approaches we applied (GLM, PA/PO model, Bayesian occupancy model, MaxEnt random background, and MaxEnt TGB) all projected an increase in probability of occurrence over time in forest set‐aside from production. All but one model (MaxEnt TGB) projected a decline in the already very low probability of occurrence in production forest. Thus, the range of modeling approaches applied here produced concurrent forest management conclusions, highlighting the importance of set‐aside forests for the persistence of *P. ferrugineofuscus*. Our results demonstrate that CSD can be a useful forecasting resource, with the potential to reliably inform land management and conservation decision making.

All models based on CSD achieved good spatial fit and predicted distribution maps indicated agreement that central Sweden was the most suitable for *P. ferrugineofuscus*. Nevertheless, there was quantitative variation among model forecasts. Thus, model performance may vary depending on whether it is assessed spatially or temporally (Smith et al., 2013). The MaxEnt models projected the smallest amount of change over time and, in particular, the TGB method failed to capture the decline in suitability in production forest that was projected by all other models. Previous work has found that, for spatially biased data in MaxEnt, selecting background points (sometimes referred to as “pseudo‐absences”) based on the presence of other ecologically similar species (the target‐group background (TGB) method) resulted in better model performance than taking a random background sample (Phillips et al., 2009); therefore, the poorer performance of the TGB approach was unexpected. The TGB model estimated a weaker effect of spruce volume on species occurrence compared to the other models, which may explain the differing projection trends. It is likely therefore that the selection of species for the TGB sample is important in determining model performance. Moreover, our results demonstrate that previously tested methods to reduce problems of spatial recording bias are not necessarily universally applicable (Stolar & Nielsen, 2015). Thus, the comparison of multiple different models in order to establish agreement has the potential to improve reliability and is likely to be of particular importance when extending studies to new regions and species.

Previous work has suggested that, in order to improve forecasting, variation among models can be dealt with by using an ensemble approach (Araújo & New, 2007; Marmion, Parviainen, Luoto, Heikkinen, & Thuiller, 2009). Indeed, averaging across projections from the models based on CSD resulted in forecasts of relative change that were quantitatively similar to forecasts from the colonization–extinction model. Nevertheless, overall the models based on CSD tended to overpredict increases in set‐aside forests and underpredict declines in production forest compared to the colonization–extinction model based on systematically collected data. By capturing the slow dynamics of certain species, colonization–extinction models are expected to yield more informative predictions of species occurrences than static SDMs (Yackulic, Nichols, Reid, & Der, 2015). Data on species dynamics are rare, however, and our results show that similar qualitative conclusions can be reached using occurrence models based on widely available citizen science occurrence data.

The use of presence–absence, rather than presence‐only, data is often considered preferable for species distribution modeling (Brotons, Thuiller, Araújo, & Hirzel, 2004). Our results support this assertion as the models which used presence–absence data (GLM and PA/PO model) projected larger declines in production forest, which were more acquiescent with the colonization–extinction model forecasts. Our results additionally support the PA/PO model (Fithian et al., 2015) as a promising advance in the efficient use of available data, due to the good performance demonstrated here and the requirement for only a small amount of presence–absence data. Obtaining presence–absence data for this study was a time‐consuming but worthwhile endeavor, as the use of presence–absence data avoids recording biases being modeled as species’ habitat associations (Yackulic et al., 2013). However, this also highlights the benefit of asking citizen scientists to provide information on their methodologies during data uploading. A slight increase in information provided can greatly improve the value of ad hoc observation data; for example, complete species lists can be used to ascertain absences (Isaac et al., 2014).

Occupancy modeling has been advocated as a particularly useful tool for extracting robust conclusions from citizen science data (Bird et al., 2014). We applied presence‐only data to the occupancy framework, which is a relatively novel approach (but see Kéry, Royle, et al. (2010) and van Strien, Termaat, Groenendijk, Mensing, and Kery (2010) for early examples). Previous work has found that species lists must be comprehensive in order to produce reliable trends (van Strien et al., 2010). However, based on our results, we suggest that both short and long species lists can be used together, along with an informative detectability variable reflecting recorder experience, in order to make use of all available observation data. One limitation of our approach was that the occurrence of the focal species was modeled relative to a wider group of ecologically similar species. As a result, our predictions were of the occurrence of *P. ferrugineofuscus* given the presence of other old‐forest indicator fungi, which explains the high probabilities of occurrence in the predicted distribution maps. Nevertheless, projections of relative change were reasonable, suggesting that reliable results can be obtained even for spatially biased data, supporting conclusions by Higa et al. (2015).

Of importance in generating reasonable projections was the inclusion of mechanistic assumptions. The incorporation of mechanistic assumptions into correlative models can provide novel insights into the processes affecting species dynamics (Swab et al., 2015). The incorporation of mechanistic assumptions here improved the biological realism of the models, by capturing aspects of *P. ferrugineofuscus* ecology which were not included in the correlative structures and reducing the likelihood of overpredicting species occurrence.

This study is one of the few to apply species distribution models to CSD for a sessile species (but see Marmion et al. (2009) for a study on plants). Deadwood‐dependent fungi are a less well‐studied organism group relative to the popular birds and butterflies; however, such sessile species could in fact be particularly appealing for citizen science initiatives, given the opportunity for time to be taken over identification. Moreover, deadwood‐dependent fungi are a functionally very important group (Ottosson et al., 2015), and their successful modeling could facilitate the consideration of different facets of ecosystem functioning in forest forecasting. For example, *P. ferrugineofuscus* is a red‐listed species and its presence is likely to indicate a relatively natural forest and the presence of other deadwood (spruce)‐dependent species. The results presented here open up the opportunity for CSD on other sessile organism groups, such as lichens and bryophytes, to also be used in modeling and forecasting.

We have shown that models based on citizen science data projected trends in *P. ferrugineofuscus* occurrence in response to forest management that were congruent with trends from a model based on systematically collected field data on colonization–extinction events. Applying a range of approaches based on different assumptions and achieving agreement among them strengthened confidence in the results. Citizen science data hold the potential to be reliably applied in forecasting species responses to land use scenarios, opening up the possibility that such extensive data could be useful for conservation and forest management planning.

## Conflict of Interest

None declared.

## Data Accessibility

Species observation data are available from the Swedish Lifewatch website; www.analysisportal.se. National forest data, “kNN‐Sweden,” are available from http://skogskarta.slu.se. The EURO4M Mesan dataset (climate data) is publicly available through the Earth System Grid Federation (ESGF), for example, http://esg-dn1.nsc.lui.se and search from “mesan.”
