# Supplementary material for: Evaluating citizen science data for forecasting species responses to national forest management
Source: Ecol Evol. 2016 Dec 20;7(1):368–78. doi: 10.1002/ece3.2601 (PMC5216679; doi:10.1002/ece3.2601)
Supplement: Supplementary file 1 [file ECE3-7-368-s001.docx]

**Supporting Information**

**Appendix S1: Establishing a presence-absence dataset**

In order to obtain a presence-absence dataset for *Phellinus ferrugineofuscus* we conducted interviews of 10 volunteer recorders. Recorders were initially selected by identifying individuals who had submitted a large number of records of wood-living fungi (in terms of a unique combination of date and location, thus spatial and temporal coverage varied amongst recorders). We also aimed to achieve a good spatial coverage of records, spanning the full latitudinal and longitudinal extent of the study area as far as possible.

Recorders were asked explicit questions about their methods when recording in the field. Questions were framed around four main points:

1. Is the recorder able to identify the ten species of interest (*Amylocystis lapponica*, *Fomitopsis pinicola*, *Fomitopsis rosea, Leptoporus mollis*, *Phellinus chrysoloma*, *Phellinus ferrugineofuscus*, *Phellinus nigrolimitatus*, *Phellinus viticola*, *Phlebia centrifuga* and *Trichaptum abietinum*)?
2. Does the recorder always report these ten species when found?
3. Does the recorder work alone or in a group, and does this affect their search methods and reporting?
4. Has the recorder’s behaviour changed over time?

Of the 10 recorders interviewed, we established that eight used methods that were consistent and rigorous enough for their observation data to be used as presence-absence data. However, it became clear that three of the species (*F. pinicola*, *P.viticola* and *T. abietinum*) were not regularly reported due to their commonness (and therefore lack of interest to the recorders). For those recorders who did not regularly report these three species (three out of the selected eight recorders), we treated the observations of these species as missing data. For the remaining recorders the data on these species were maintained.

These data were combined with a further source of presence-absence data. During the period 2008-2013, species surveys were carried out by thirty people with the purpose of providing information on species’ status for Red Listing. In these data, the three common species (*F. pinicola*, *P. viticola* and *T. abietinum*) were again not reported reliably, but data for the remaining seven species was rigorously collected.

Combining these data gave a total of 15,508 grid cells (100 m resolution) with presence-absence observation data. The locations of these cells in Sweden are shown in Fig. S1.


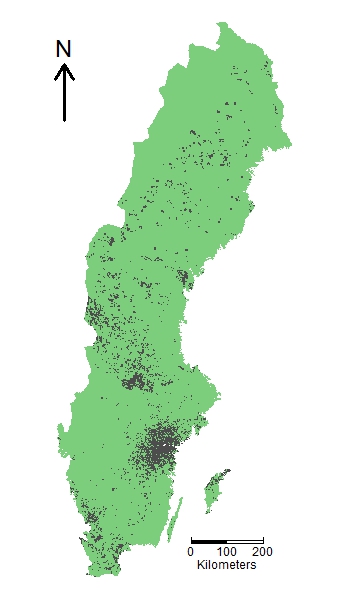


**Figure S1**. The location of the 15,508 grid cells (100 m resolution) with presence-absence data on *Phellinus ferrugineofuscus* for 2000-13.

**Appendix S2: Environmental data**

**A. kNN forest data**

Forest stand variables were based on estimates which combine satellite images and ground-truthing; ‘kNN-Sweden’ (http://skogskarta.slu.se; Reese *et al.*, 2003). Data for living spruce volume and forest stand age were obtained at 25 m grid cell resolution for the years 2000, 2005 and 2010. The data were aggregated to 100 m grid cell resolution by taking the maximum value. The maximum was used because *P. ferrugineofuscus* occurrence is expected to increase with increasing forest age and spruce volume (Nordén *et al.*, 2013). We temporally matched the forest data to the species’ observation data.

**B. Connectivity calculation based on kNN data**

We used the kNN data to calcualte connectivity to old forest, which reflects the potential dispersal sources for the species in the surrounding landscape. We first selected 25 m grid cells with a forest age >100 years. We then aggregated the living spruce volume to the 500 m grid cell resolution by summing the volume in these ‘old forest’ cells. Finally, we divided by 400 to give the mean volume per hectare. This coarse resolution increased computational efficiency. The distances from the focal cell to all 500 m grid cells within a 20 km buffer were found, and the connectivity S_i_ of the focal cell *i* was calculated as

S_i_ = Σ*_j_*_≠_*_i_* v_j_*exp(-α d_ij_),

where α is the dispersal parameter, d_ij_ is the distance in km from focal cell *i* to cell *j*, and *v* is the mean volume of spruce in cell *j* (adapted from Nordén *et al.*, 2013). There is limited information available on species’ dispersal distances therefore we tested three values for the dispersal parameter (α = 1, 0.2, 0.1) representing a mean dispersal distance of 1 km, 5 km and 10 km, respectively.

**C. Climate data**

Gridded meteorological observations of daily precipitation and daily mean temperature for the period 1989-2010 were obtained from the EURO4M Mesan dataset (Landelius *et al.*, 2016) at a spatial resolution of 6.25 km x 6.25 km (0.05 latitude x 0.05 longitude). The high resolution gridding procedure is based on optimum interpolation using a large database of observational data together with gridded data at coarser resolution (0.2 latitude x 0.2 longitude) from a regional reanalysis (Dahlgren *et al.*, 2016). The gridding procedure is based on optimum interpolation procedure (Johansson, 2000; Johansson & Chen, 2003, 2005). Mean annual temperature was calculated from the daily mean for 1989-2010. Seasonally accumulated precipitation was calculated from May to November, and then averaged over the period 1989-2010. This timeframe includes the ten years prior to the species observation data as fruiting bodies observed from 2000 onwards may reflect colonisation several years earlier.

**D. Explanatory covariates derived from digital elevation maps**

A wetness index was calculated from a 50 m resolution digital elevation map (DEM) of Sweden (Swedish land survey service; www.lantmateriet.se) using ArcMap version 10.2.2. Wetness was calculated as

log(FlowAccumulation/Width) * 100 / (cosine * Slope),

where *flow accumulation* was calculated using the hydrology tool in ArcMap, *width* was derived from cell aspect, and *slope* was calculated as percent rise using the slope tool.

The second environmental variable derived from the DEM was created to reflect the steepness and orientation of a cell. First, cell aspect was multiplied by cosine to give the north-south orientation of the cell (indicating sun exposure), and this was then multiplied by the cell slope. Both covariates were then aggregated to the 100 m grid cell resolution by taking the mean.

**Appendix S3: Old-forest indicator species**

**Table S1**. Old-forest indicator species (N=35; Nitare, 2000). The detection of these species was used to indicate the non-detection of our focal species, *Phellinus ferrugineofuscus*, from a cell in the data applied to the occupancy modelling. Species names correspond to those currently recommended by the Swedish Taxonomic Database (www.dyntaxa.se) and are the same as used by Swedish LifeWatch.

| Species |
| --- |
| *Amylocystis lapponica* (Romell) Singer |
| *Antrodia albobrunnea* (Romell) Ryvarden |
| *Antrodia pulvinascens* (Pilat) Niemelä |
| *Cinereomyces lenis* (P. Karst.) Spirin |
| *Climacocystis borealis* (Fr.) Kotl. & Pouzar |
| *Dichomitus campestris* (Quél.) Domanski & Orlicz |
| *Fistulina hepatica* (Schaeff.: Fr.) With. |
| *Fomitopsis rosea* (Alb. & Schwein.:Fr.) P.Karst. |
| *Grifola frondosa* (Dicks.:Fr.) Gray |
| *Hapalopilus croceus* (Fr.) Donk |
| *Haploporus odorus* (Sommerf.: Fr.) Bondartsev & Singer |
| *Haploporus tuberculosus* (Fr.) Niemelä & Y.C. Dai |
| *Inonotus dryadeus* (Pers. : Fr.) Murrill |
| *Inonotus rheades* (Pers.) P. Karst. |
| *Junghuhnia collabens* (Fr.) Ryvarden |
| *Leptoporus mollis* (Pers.:Fr.) Quél. |
| *Meruliopsis taxicola* (Pers.: Fr.) Bondartsev |
| *Oligoporus guttulatus* (Peck) Gilb. & Ryvarden |
| *Oligoporus lateritius* (Renv.) Ryvarden & Gilb. |
| *Onnia leporina* (Fr.) H. Jahn |
| *Onnia tomentosa* (Fr.) P. Karst. |
| *Osmoporus protractus* (Fr.) Bondartsev |
| *Oxyporus corticola* (Fr.) Ryvarden |
| *Perenniporia subacida* (Peck) Donk |
| *Phaeolus schweinitzii* (Fr.: Fr.) Pat. |
| *Phellinus chrysoloma* (Fr.) Donk |
| *Phellinus ferruginosus* (Schrad.: Fr.) Pat. |
| *Phellinus nigrolimitatus* (Romell) Bourdot & Galzin |
| *Phellinus pini* (Brot.:Fr.) A.Ames |
| *Phellinus populicola* Niemelä |
| *Phellinus viticola* (Schwein. ex Fr.) Donk |
| *Phlebia centrifuga* (P. Karst) |
| *Pycnoporellus fulgens* (Fr.) Donk |
| *Skeletocutis odora* (Peck ex Sacc.) Ginns |
| *Trichaptum laricinum* (P. Karst.) Ryvarden |

**Appendix S4: Detectability variables**

Detectability is the likelihood that the target species will be both found and recorded by a citizen scientist on sites where it occurs. Detectability may vary among sites, over time and/or among recorders. If heterogeneous detection rates are expected, then identifying informative detectability variables is important for obtaining accurate estimates of species occurrence (MacKenzie *et al.*, 2003). We identified potential explanatory variables for detectability based on the existing literature and our knowledge of the citizen science dataset. In the following text, the biological reasoning behind the appropriateness or inappropriateness of each variable is explained.

***i) List length***

List length is the number of species recorded during a particular recording event. It can be treated categorically (e.g. van Strien *et al.*, 2013) or as a continuous monotonic function (e.g. Isaac *et al.*, 2014). In previous studies list length has been assumed to reflect the amount of effort expended, with a longer list length indicating a more thorough search (van Strien *et al.*, 2013). This is a reasonable assumption when species richness is generally expected to be high (or at least relatively consistent) across sites and visits. However, this reasoning does not apply well to the dataset used in this study, particularly in the case of assuming a continuous monotonic function. Wood-living fungi species richness is expected to vary amongst sites depending on forest characteristics such as forest age and substrate volume (Nordén *et al.*, 2013; Peltoniemi *et al.*, 2013). As a result, we considered it likely that list length was correlated with both recording effort and fungal species richness and therefore would not be an appropriate explanatory variable.

***ii) Day of the year***

Day of the year has been used in previous studies as it is expected that for taxa such as butterflies, higher abundances during the peak flight period will increase the detectability of the species (e.g. van Strien *et al.*, 2011; Zipkin *et al.*, 2012; Strebel *et al.*, 2014). Such an expectation is not applicable here because *P. ferrugineofuscus* is a species with fruiting bodies that are visible throughout the year and can remain identifiable for several years.

***iii) Number of days spent recording***

We identified the total number of days (during the study period 2000-2013) for which each unique recorder uploaded species observation data for the 35 old-forest indicator species as a potential explanatory variable for detectability. Recorders who submitted observations over a larger number of days have more opportunity to hone identification skills and become more efficient in the field. This variable should be independent of any variable affecting species occurrence and was therefore selected for inclusion in the final model.

The number of days spent recording was calculated based on the individual recorder’s name or the collection of names in a group of people who recorded together. Thus the same person may have been present in multiple different groups, and each of these groups was treated as a ‘unique’ recorder. This was considered a reasonable methodology as we expect the efficiency of a group to vary depending on its composition.

**Appendix S5: BUGS code for occupancy model**

The following BUGS code was used for the final occupancy model where observations of *P. ferrugineofuscus* detection were available for cell *i* and visit *j*. An explanation of model parameter denotation is given below.

model

{

## Prior distributions

adet ~ dnorm(0, 0.01)

aocc ~ dnorm(0, 0.01)

bgran ~ dnorm(0, 0.01)

bconn ~ dnorm(0, 0.01)

btemp ~ dnorm(0, 0.01)

bprec ~ dnorm(0, 0.01)

btwi ~ dnorm(0, 0.01)

dnd ~ dnorm(0, 0.01)

dnds ~ dnorm(0, 0.01)

## Loop through cells (*ncells* gives number of cells)

for (i in 1:ncells) {

## True occurrence model

z[i] ~ dbern(psi[i])

logit(psi[i]) <- aocc + bgran*gran[i] + bconn*conn[i] + btemp*temp[i] + bprec*prec[i] + btwi*twi[i]

## Loop through visits (*spi* gives number of visits per cell)

for (j in 1:spi[i]) {

## Observed data as a function of true occurrence*detection

y[i,j] ~ dbern(eff.p[i,j])

eff.p[i,j] <- z[i] * p[i,j]

## Detection model

logit(p[i,j]) <- adet + dnd*ND[i,j] + dnds*NDS[i,j]

}

}

}

Model parameter denotation:

adet = intercept parameter for detection model

aocc = intercept parameter for occurrence model

bgran = slope parameter for the relationship with living spruce

bconn = slope parameter for the relationship with connectivity

btemp = slope parameter for the relationship with temperature

bprec = slope parameter for the relationship with precipitation

btwi = slope parameter for the relationship with wetness index

dnd = slope parameter for the relationship with number of days recorded (per unique recorder)

dnds = slope parameter for the quadratic relationship with number of days recorded (per unique recorder)

gran = living spruce volume

conn = connectivity (alpha = 0.1)

temp = mean annual temperature

prec = summed precipitation May-November

twi = wetness index

DND = number of days recorded (per unique recorder)

DNDS = quadratic of number of days recorded (per unique recorder)

**Appendix S6: Forest projection data**

Forest projection data were available from the Swedish nationwide Forest Impact Analyses 2015 (SKA 15; Claesson *et al.*, 2015; Eriksson *et al.*, 2015). Projections were made for the National Forestry Inventory (NFI) plots (Fridman *et al.*, 2014) and initialized from the state observed in 2008-2012. We used projection data for a total of 17,383 NFI plots located within the boreal region of Sweden. Data on projected changes in living and dead wood spruce volume and forest age were available for each plot for every fifth year from 2020 to 2110. We used projections of the living spruce volume and forest age to calculate connectivity (for details see Appendix S7). The initial amounts and properties of dead wood on the NFI plots were based on the observed state in 2008-2012. We wrote our own software to simulate the decomposition of the dead wood on the NFI plots using the one-time chronosequence method described in Harmon *et al.*, 2000.

For the SKA 15 projections the RegWise software (version 2.2) was used. RegWise is a software component within the Heureka suite of forest decision support tools (Wikström *et al.*, 2011). The core of Heureka is made up of empirical individual tree growth (see Fahlvik *et al.*, 2014), ingrowth (Wikberg, 2004) and mortality (Elfving, 2014) models simulating tree layer development in five year time steps. In RegWise forest management actions are steered by a rule-based simulation framework. The harvest level – and consequently the development of the forest – is controlled by stated management programmes (silviculture and harvest activities) and the actual growth in each time period. Different management programmes are stated for, e.g., different forest owner categories, tree species mixtures and site factors, in accordance with what was observed at the NFI plots.

In SKA 15 different scenarios were applied concerning the intensity in forestry (harvest level) and the forest area set-aside from timber production for nature conservation purposes. Here we used a scenario which assumes that 84% of the land is used for wood production and 16% is set-aside from forestry over the coming 100 years. Tree retention practices at final felling were also included (Roberge *et al.*, 2015).

**Appendix S7: Calculating connectivity from forest projection data**

The forest projection data were used to project connectivity. Projection data were available for the NFI plots. The data are effectively a point-pattern distributed across the landscape, in contrast to the connectivity variable used in model fitting which was derived from 25 m resolution raster data. We therefore developed a method to incorporate the information on landscape structure held in the kNN forest data with the forest projection data. Here we describe the method and provide a schematic diagram in Fig S3.

We first created a 500 m resolution raster using the kNN data to indicate what proportion of each cell was ‘land’ in order to account for the spatial distribution of waterbodies, urban areas and coastlines (Fig S3a). We then identified the nearest NFI tract (a tract is a cluster of NFI plots which are surveyed together) to each grid cell, and used this tract to inform the cell values (Fig S3b). The proportion of plots on each tract which contained old forest (>100 years) was used as the probability that the 500 m grid cell contained old forest. A random draw was taken from this probability and the cell was assigned as either contains or does not contain old forest (Fig S3c). If the cell contained old forest (>100 years), then the volume of living spruce in that cell was calculated as

( proportion old forest * volume of spruce ) * proportion land

where *proportion old forest* is the proportion of plots on the tract that contain old forest, *volume of spruce* is the mean volume of spruce from plots on the tract that contain old forest, and *proportion land* is the proportion of the cell that was determined to be land (from the kNN data; depicted in Fig S3). This process created a distribution of old forest that was as spatially structured as possible given the forest projection data. We then calculated distances from the focal tract to all 500 m grid cells within a 20 km buffer and calculated connectivity using the equation given in *Environmental data* in the main text. This process was repeated for each year that the forest projection data were available.


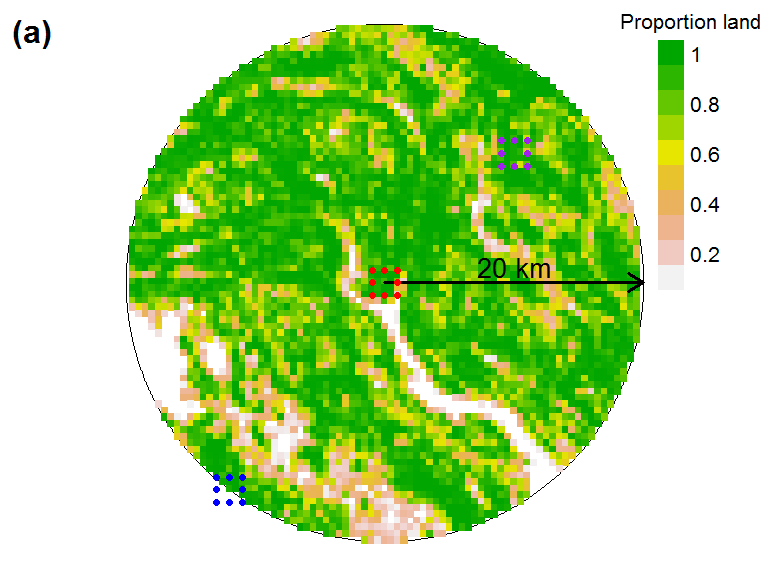


**The proportion of each 500 m cell that is ’land’ is identified.**

The red, purple and blue points are NFI tracts (a tract is a cluster of NFI plots which are surveyed together). The red tract is the focal tract in this example, and connectivity calculations are carried out on cells within a 20 km buffer of this focal tract.

**The nearest NFI tract to each grid cell is identified.**

Here the cells are colour-coded to indicate the nearest tract. The forest projection data for the red tract will inform the values assigned to the pink cells (and so on with the blue and purple tracts).


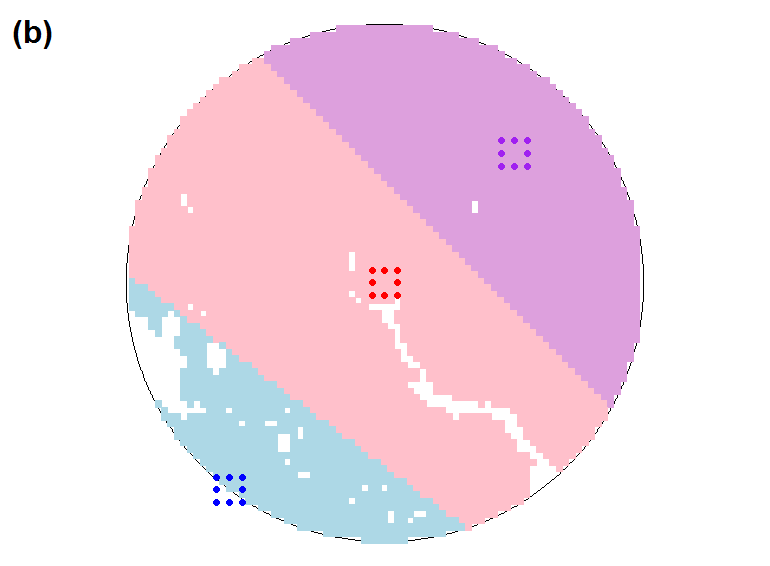


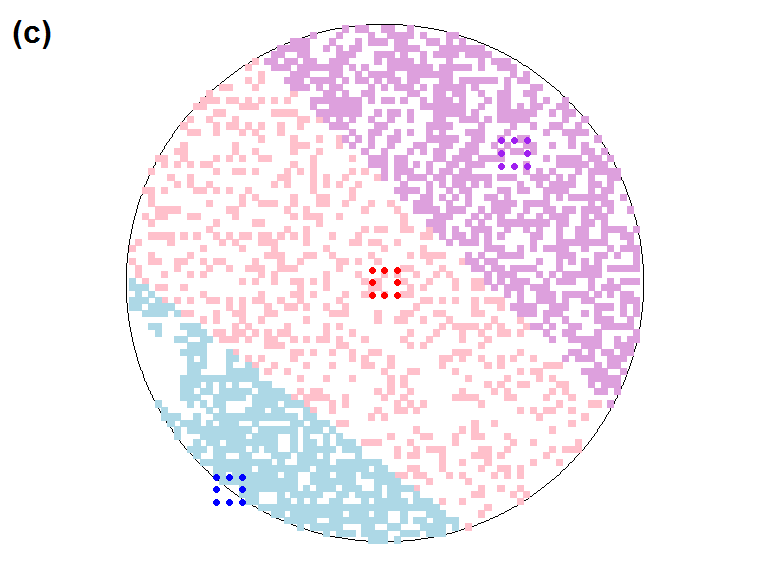


**Cells are assigned as either contains or does not contain old forest.**

In this example, a quarter of the plots on the red tract were projected to contain old forest, therefore the pink cells had a probability of 0.25 of containing old forest. For each pink cell, a random draw was taken from this probability in order to assign the presence/absence of old forest.

**Figure S3**. Schematic detailing three stages of the method for calculating connectivity from the projection data. The full process is described in detail in the text.

**Appendix S8: Mechanistic assumptions**

Mechanistic assumptions were incorporated into the projections of *P. ferrugineofuscus* occurrence. Firstly, the species could not occur where no dead spruce wood was present. This assumption is easily justifiable given the species is dead-wood dependent. Secondly, the species could not occur where forest stand age was >25 and <64 years. This assumption was incorporated based on knowledge of the species’ ecology, and supported by observations from the systematically collected field data on *P. ferrugineofuscus* colonisation-extinction dynamics. Firstly, the youngest non-cut forest that the species was recorded in the systematically collected data was 64 years. This assumption was substantiated using the citizen science observations of the species; it is clear from Fig. S4 that *P. ferrugineofuscus* occurs very rarely in forest stands younger than 64 years. Secondly, the species was not excluded from forest ≤25 years because some fruiting bodies may persist after the forest stand is cut if there is some dead wood retention. Any such retained dead wood is likely to have decomposed after around 25 years and no longer be suitable for *P. ferrugineofuscus*.


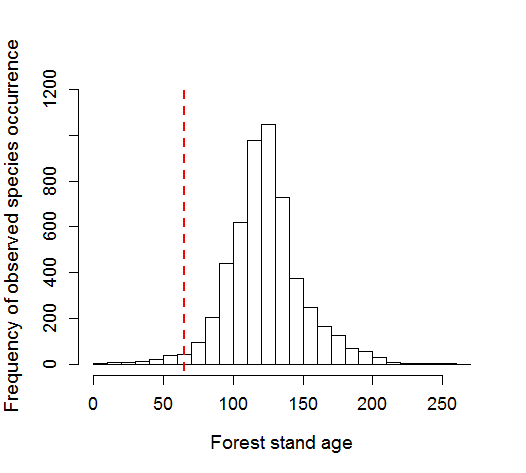


**Figure S4**. Histogram of forest stand age in the 100 m grid cells where *Phellinus ferrugineofuscus* was observed in 2000-13 according to the presence-only citizen science data. The dashed red line indicates forest age = 64 years.

**Appendix S9: Block-cross validation**

We assessed the spatial accuracy of model predictions using the presence-absence dataset (see *Species observation data* in main text). We used block cross-validation to assess model predictive ability at the 100 m grid cell resolution (as recommended by Merow *et al.*, 2014). We divided the landscape into 50 km grid cells. A randomly selected 80% of these 50 km cells were used as training data for model fitting, and the withheld 20% were used for model testing. Models fitted to the training data were used to predict the probability of species occurrence at the presence-absence data points falling within the withheld 50 km grid cells. We then applied the forest age mechanistic assumption (see *Colonisation-extinction model based on systematically collected field data* in main text), but given the lack of national GIS data on dead wood volume, we could not apply the assumption relating to dead wood presence. We then calculated the area under the receiver operating curve (AUC; Fielding & Bell, 1997). AUC scores range from zero to one, with a score of greater than 0.5 indicating better discrimination ability than expected at random, while a score greater than 0.7 is indicates reasonable discrimination ability and greater than 0.9 indicates very good discrimination ability (Pearce & Ferrier, 2000). This process was carried out five times, such that each data point was used in model testing only once. Results showed that all models achieved good predictive accuracy (Fig. S5).


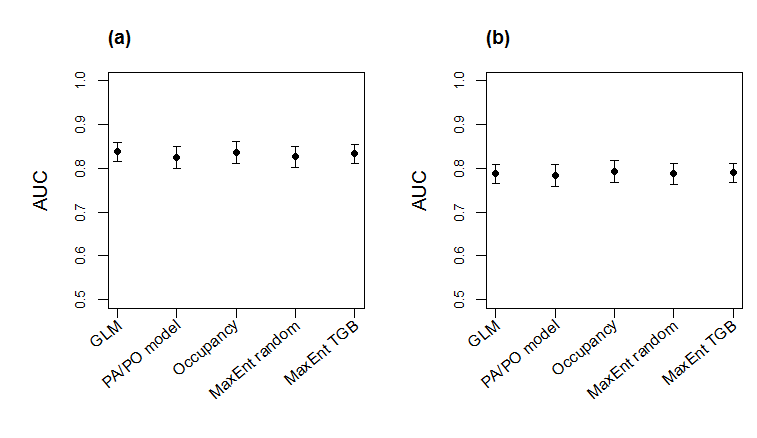


**Figure S5**. Mean AUCs ±SD calculated on (a) training data, and (b) withheld testing data, for each model based on citizen science data, obtained from five-fold block cross-validation.

**Appendix S10: Final models**

Details of final models including all parameter estimates (Table S2), fitted lines for the four most important explanatory variables (Fig S5), and a sensitivity analysis of the effect on model projections of incorporating mechanistic assumptions (Fig S6).

**Table S2.** Details of the citizen science data models used for making projections.

1. GLM

| Variable | Parameter estimate | Std Error | Z value | *P* |
| --- | --- | --- | --- | --- |
| Intercept | -2.939 | 0.045 | -65.90 | <0.001 |
| Living spruce volume | 0.773 | 0.035 | 22.07 | <0.001 |
| Connectivity (α = 0.1) | 0.720 | 0.031 | 23.21 | <0.001 |
| Temperature | -0.352 | 0.053 | -6.68 | <0.001 |
| Precipitation | -0.093 | 0.045 | -2.07 | 0.04 |
| Temperature * precipitation | -0.234 | 0.050 | -4.69 | <0.001 |

1. PA/PO model

| Variable | Parameter estimate | Std Error | Z value | *P* |
| --- | --- | --- | --- | --- |
| Environmental model |  |  |  |  |
| Intercept | -3.654 | 0.033 | -109.74 | <0.001 |
| Living spruce volume | 0.907 | 0.013 | 72.01 | <0.001 |
| Connectivity (α = 1) | 0.241 | 0.008 | 29.83 | <0.001 |
| Temperature | -0.929 | 0.021 | -44.91 | <0.001 |
| Precipitation | -0.254 | 0.018 | -13.99 | <0.001 |
| Temperature * precipitation | -0.379 | 0.022 | -17.32 | <0.001 |
| Bias model |  |  |  |  |
| Population density | 0.267 | 0.020 | 13.03 | <0.001 |
| Population density^2 | -0.387 | 0.056 | -6.97 | <0.001 |
| Distance to small roads | -0.189 | 0.032 | -5.84 | <0.001 |
| Distance to small road^2 | -2.439 | 0.141 | -17.35 | <0.001 |

1. Occupancy model

|  | Statistics on the posterior distribution of parameters | | | |
| --- | --- | --- | --- | --- |
| Variable | Mean | Std Deviation | 2.5% quantile | 97.5% quantile |
| Occurrence model |  |  |  |  |
| Intercept | -0.689 | 0.073 | -0.827 | -0.542 |
| Living spruce volume | 0.752 | 0.041 | 0.675 | 0.835 |
| Connectivity (α = 0.1) | 0.544 | 0.035 | 0.478 | 0.615 |
| Precipitation | -0.082 | 0.026 | -0.133 | -0.031 |
| Temperature | -0.240 | 0.028 | -0.296 | -0.185 |
| Wetness index | 0.076 | 0.022 | 0.032 | 0.120 |
| Detection model |  |  |  |  |
| Intercept | -0.106 | 0.075 | -0.250 | 0.045 |
| Number of days recorded | 0.080 | 0.035 | 0.012 | 0.150 |
| Number of days recorded^2 | 0.063 | 0.025 | 0.015 | 0.114 |

1. MaxEnt - random background points

| Variable | Lambda | Contribution | Permutation importance |
| --- | --- | --- | --- |
| Living Spruce volume | 5.931 | 42.97 | 56.49 |
| Connectivity (α=1) | 2.063 | 37.54 | 4.13 |
| Precipitation | -2.513 | 2.32 | 5.36 |
| Temperature | -4.182 | 14.64 | 27.27 |
| Precipitation * temperature | -3.884 | 0.02 | 6.64 |
| Wetness index | -0.474 | 0.02 | 0.12 |

1. MaxEnt – TGB (wood living fungi)

| Variable | Lambda | Contribution | Permutation importance |
| --- | --- | --- | --- |
| Living Spruce volume | 2.955 | 23.72 | 41.62 |
| Connectivity (α=0.1) | 1.852 | 70.12 | 36.41 |
| Precipitation | -0.251 | 0.00 | 0.56 |
| Temperature | -1.349 | 5.60 | 20.82 |
| Wetness index | 0.523 | 0.16 | 0.59 |


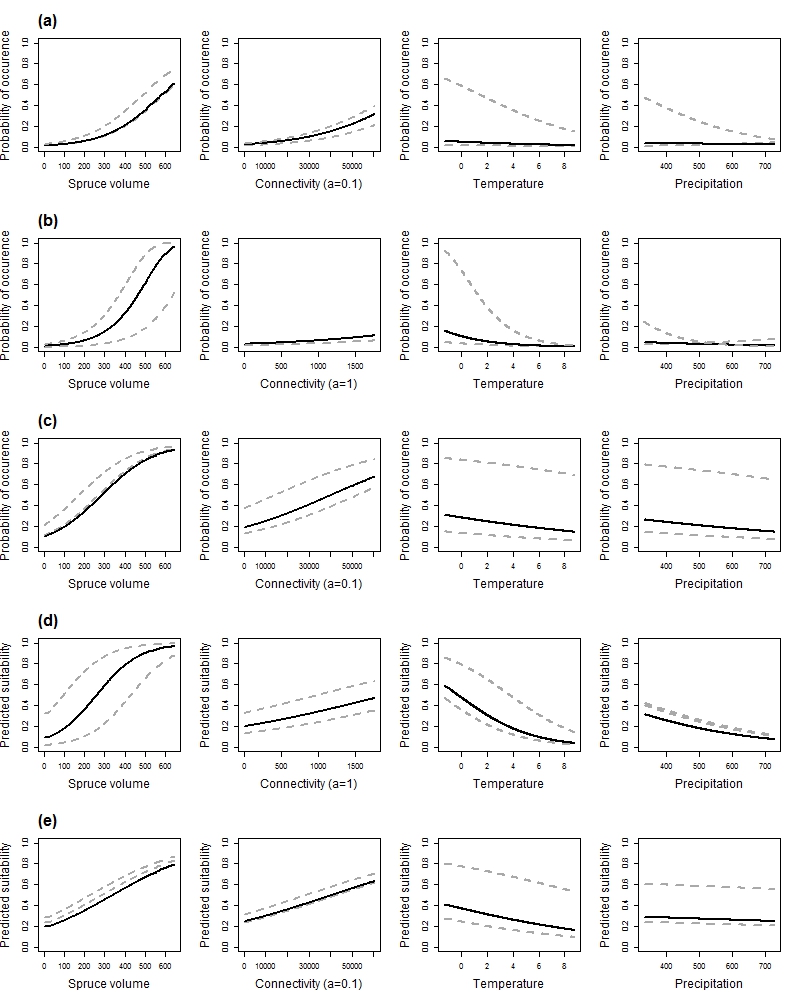


**Figure S6**. Fitted lines for the most important explanatory variables in the (a) GLM, (b) PA/PO model, (c) occupancy model, (d) MaxEnt random background, and (e) MaxEnt TGB. All variables other than that on the x-axis are kept constant at the median (solid black line), 10^th^ percentile or 90^th^ percentile (dashed grey lines) as observed in the presence-absence data.


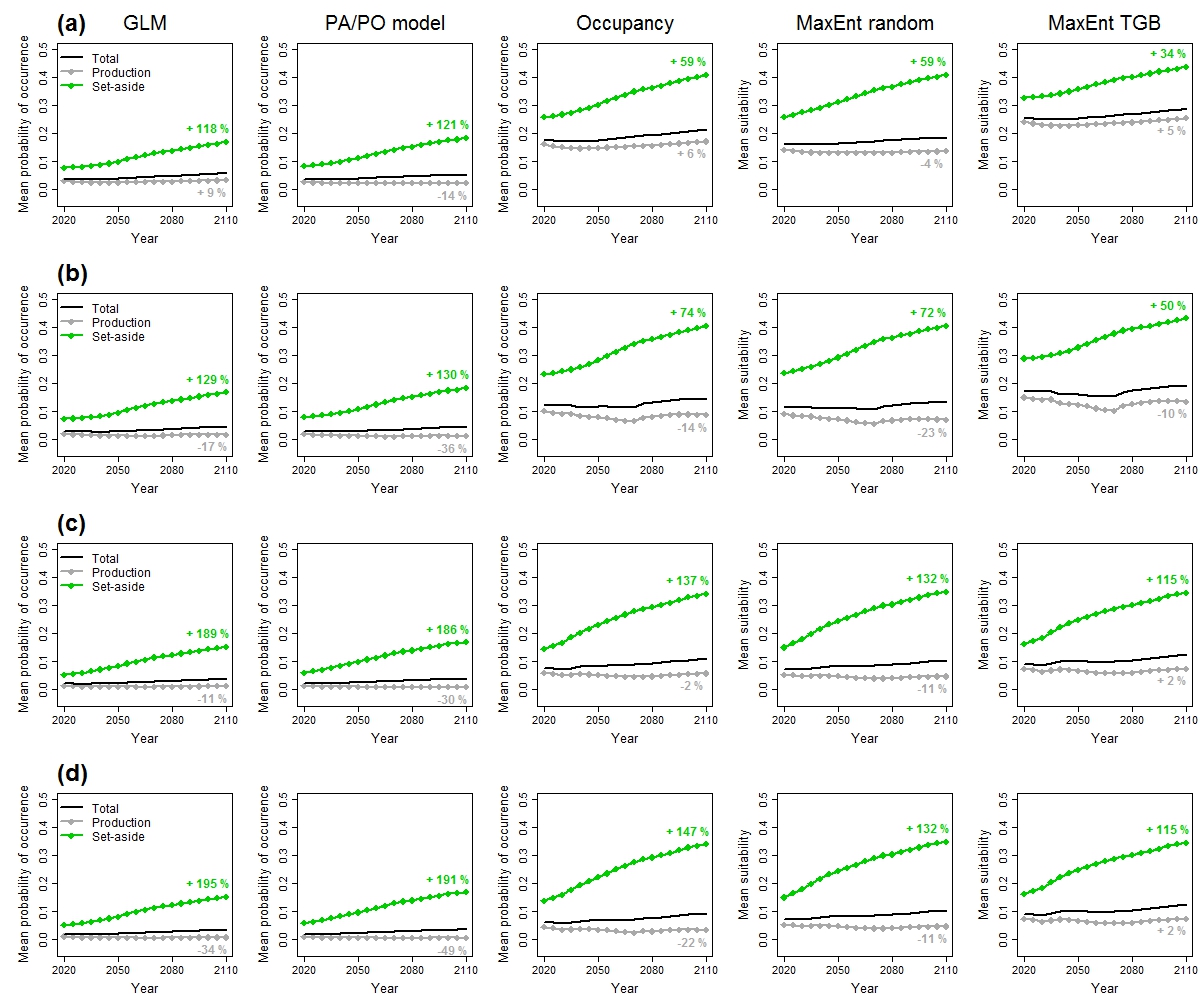


**Figure S7.** Projections of mean probability of *Phellinus ferrugineofuscus* occurrence (or suitability) in response to projected forest management when: (a) no mechanistic assumptions are applied; (b) the forest age threshold mechanistic assumption is applied (the species cannot occur when forest age is >25 and <64 years); (c) the dead wood mechanistic assumption is applied (the species cannot occur where no dead wood is present); and (d) both the age threshold and dead wood mechanistic assumptions are applied (as presented in the main text). The relative changes in projected probability of occurrence (%) from 2010 to 2110 are given for the total, and for set-aside and production forest separately.

**References**

Claesson, S., Duvemo, K., Lundström, A. & Wikberg, P.E. (2015) Forest Impact Analysis 2015 - SKA 15 (Skogliga konsekvensanalyser - SKA 2015). In. Swedish Forest Agency, Report 10.

Dahlgren, P., Landelius, T., Kållberg, P. & Gollvik, S. (2016) A high resolution regional reanalysis for Europe Part 1: 3-dimensional reanalysis with the regional High Resolution Limited Area Model (HIRLAM). *Quarterly Journal of the Royal Meteorological Society*,

Elfving, B. (2014) Modelling of natural mortality in Heureka (Modellering av naturlig avgång i Heureka). In. SLU, Dept. of forest ecology and management, Memorandum March 17.

Eriksson, A., Snäll, T. & Harrison, P.J. (2015) Analys av miljöförhållanden - SKA 15. In. Swedish Forest Agency, Report 11.

Fahlvik, N., Elfving, B. & Wikström, P. (2014) Evaluation of growth functions used in the Swedish Forest Planning System Heureka. *Silva Fennica*, **48**, Article ID 1013.

Fielding, A.H. & Bell, J.F. (1997) A review of methods for the assessment of prediction errors in conservation presence/absence models. *Environmental Conservation*, **24**, 38-49.

Fridman, J., Holm, S., Nilsson, M., Nilsson, P., Ringvall, A.H. & Ståhl, G. (2014) Adapting National Forest Inventories to changing requirements - the case of the Swedish National Forest Inventory at the turn of the 20th century. *Silva Fennica*, **48**, Article ID 1095.

Harmon, M.E., Krankina, O.N. & Sexton, J. (2000) Decomposition vectors: a new approach to estimating woody detritus decomposition dynamics. *Canadian Journal of Forest Research*, **30**, 76-84.

Isaac, N.J.B., A.J. van Strien, T.A. August, M.P. de Zeeuw, & D.B. Roy. (2014) Statistics for citizen science: extracting signals of change from noisy ecological data. Methods in Ecology and Evolution 5:1052-1060.

Johansson, B. (2000) Areal Precipitation and Temperature in the Swedish Mountains: An evaluation from a hydrological perspective. *Hydrology Research*, **31**, 207-228.

Johansson, B. & Chen, D. (2003) The influence of wind and topography on precipitation distribution in Sweden: statistical analysis and modelling. *International Journal of Climatology*, **23**, 1523-1535.

Johansson, B. & Chen, D. (2005) Estimation of areal precipitation for runoff modelling using wind data: a case study in Sweden. *Climate Research*, **29**, 53-61.

Landelius, T., Dahlgren, P., Gollvik, S., Jansson, A. & Olsson, E. (2016) A high resolution regional reanalysis for Europe. Part 2: 2D analysis of surface temperature, precipitation and wind. *Quarterly Journal of the Royal Meteorological Society. In revision.*,

MacKenzie, D.I., Nichols, J.D., Hines, J.E., Knutson, M.G. & Franklin, A.B. (2003) Estimating site occupancy, colonization, and local extinction when a species is detected imperfectly. *Ecology*, **84**, 2200-2207.

Merow, C., Smith, M.J., Edwards, T.C., Guisan, A., McMahon, S.M., Normand, S., Thuiller, W., Wüest, R.O., Zimmermann, N.E. & Elith, J. (2014) What do we gain from simplicity versus complexity in species distribution models? *Ecography*, **37**, 1267-1281.

Nitare, J. (2000) *Signalarter: indikatorer på skyddsvärd skog; flora över kryptogamer*. Skogsstyrelnsens Förlag, Jönköping.

Nordén, J., Penttilä, R., Siitonen, J., Tomppo, E. & Ovaskainen, O. (2013) Specialist species of wood-inhabiting fungi struggle while generalists thrive in fragmented boreal forests. *Journal of Ecology*, **101**, 701-712.

Pearce, J. & Ferrier, S. (2000) Evaluating the predictive performance of habitat models developed using logistic regression. *Ecological Modelling*, **133**, 225-245.

Peltoniemi, M., Penttila, R. & Makipaa, R. (2013) Temporal variation of polypore diversity based on modelled dead wood dynamics in managed and natural Norway spruce forests. *Forest Ecology and Management*, **310**, 523-530.

Reese, H., Nilsson, M., Pahlén, T.G., Hagner, O., Joyce, S., Tingelöf, U., Egberth, M. & Olsson, H. (2003) Countrywide estimates of forest variables using satellite data and field data from the national forest inventory. *Ambio*, **32**, 542-548.

Roberge, J.-M., Lämås, T., Lundmark, T., Ranius, T., Felton, A. & Nordin, A. (2015) Relative contributions of set-asides and tree retention to the long-term availability of key forest biodiversity structures at the landscape scale. *Journal of Environmental Management*, **154**, 284-292.

Strebel, N., Kéry, M., Schaub, M. & Schmid, H. (2014) Studying phenology by flexible modelling of seasonal detectability peaks. *Methods in Ecology and Evolution*, **5**, 483-490.

van Strien, A.J., van Swaay, C.A.M. & Kery, M. (2011) Metapopulation dynamics in the butterfly Hipparchia semele changed decades before occupancy declined in The Netherlands. *Ecological Applications*, **21**, 2510-2520.

van Strien, A.J., van Swaay, C.A.M. & Termaat, T. (2013) Opportunistic citizen science data of animal species produce reliable estimates of distribution trends if analysed with occupancy models. *Journal of Applied Ecology*, **50**, 1450-1458.

Wikberg, P.E. (2004) *Occurrence, morphology and growth of understory saplings in Swedish forests.* Ph.D. Thesis, Swedish University of Agricultural Sciences, Umeå.

Wikström, P., Edenius, L., Elfving, B., Eriksson, L.O., Lämås, T., Sonesson, J., Öhman, K., Wallerman, J., Waller, C. & Klintebäck, F. (2011) The Heureka Forestry Decision Support System: An Overview. *Mathematical and Computational Forestry & Natural Resource Sciences*, **3**, 87-95.

Zipkin, E.F., Grant, E.H.C. & Fagan, W.F. (2012) Evaluating the predictive abilities of community occupancy models using AUC while accounting for imperfect detection. *Ecological Applications*, **22**, 1962-1972.
